# Molecular characterization of the circadian clock in patients with Parkinson’s disease–CLOCK4PD Study protocol

**DOI:** 10.1371/journal.pone.0305712

**Published:** 2024-07-19

**Authors:** Müge Yalçin, Ana Rita Peralta, Carla Bentes, Cristiana Silva, Tiago Guerreiro, Joaquim J. Ferreira, Angela Relógio

**Affiliations:** 1 Institute for Theoretical Biology (ITB), Charité—Universitätsmedizin Berlin, Corporate Member of Freie Universität Berlin, Humboldt-Universität zu Berlin and Berlin Institute of Health, Berlin, Germany; 2 Molecular Cancer Research Center (MKFZ), Medical Department of Hematology, Oncology, and Tumour Immunology, Charité—Universitätsmedizin Berlin, Corporate Member of Freie Universität Berlin, Humboldt-Universität zu Berlin and Berlin Institute of Health, Berlin, Germany; 3 Institute for Systems Medicine and Faculty of Human Medicine, MSH Medical School Hamburg, Hamburg, Germany; 4 EEG/Sleep Laboratory, Department Neurosciences and Mental Health, Unidade Local de Saude Santa Maria—ULSSM, Lisbon, Portugal; 5 Department of Neurology, Faculdade de Medicina, Universidade de Lisboa, Lisbon, Portugal; 6 Instituto de Fisiologia, Faculdade de Medicina, Universidade de Lisboa, Lisbon, Portugal; 7 CNS-Campus Neurológico, Torres Vedras, Portugal; 8 Centro de Estudos Egas Moniz, Faculdade de Medicina, Universidade de Lisboa, Lisbon, Portugal; 9 LASIGE, Faculdade de Ciências, Universidade de Lisboa, Lisbon, Portugal; 10 Instituto de Medicina Molecular, Faculdade de Medicina, Universidade de Lisboa, Lisbon, Portugal; 11 Laboratory of Clinical Pharmacology and Therapeutics, Faculdade de Medicina, Universidade de Lisboa, Lisbon, Portugal; Dokkyo Medical University: Dokkyo Ika Daigaku, JAPAN

## Abstract

**Introduction:**

Circadian rhythms (CRs) orchestrate intrinsic 24-hour oscillations which synchronize an organism’s physiology and behaviour with respect to daily cycles. CR disruptions have been linked to Parkinson’s Disease (PD), the second most prevalent neurodegenerative disorder globally, and are associated to several PD-symptoms such as sleep disturbances. Studying molecular changes of CR offers a potential avenue for unravelling novel insights into the PD progression, symptoms, and can be further used for optimization of treatment strategies. Yet, a comprehensive characterization of the alterations at the molecular expression level for core-clock and clock-controlled genes in PD is still missing.

**Methods and analysis:**

The proposed study protocol will be used to characterize expression profiles of circadian genes obtained from saliva samples in PD patients and controls. For this purpose, 20 healthy controls and 70 PD patients will be recruited. Data from clinical assessment, questionnaires, actigraphy tracking and polysomnography will be collected and clinical evaluations will be repeated as a follow-up in one-year time. We plan to carry out sub-group analyses considering several clinical factors (e.g., biological sex, treatment dosages, or fluctuation of symptoms), and to correlate reflected changes in CR of measured genes with distinct PD phenotypes (diffuse malignant and mild/motor-predominant). Additionally, using NanoString^Ⓡ^ multiplex technology on a subset of samples, we aim to further explore potential CR alterations in hundreds of genes involved in neuropathology pathways.

**Discussion:**

CLOCK4PD is a mono-centric, non-interventional observational study aiming at the molecular characterization of CR alterations in PD. We further plan to determine physiological modifications in sleep and activity patterns, and clinical factors correlating with the observed CR changes. Our study may provide valuable insights into the intricate interplay between CR and PD with a potential to be used as a predictor of circadian alterations reflecting distinct disease phenotypes, symptoms, and progression outcomes.

## Introduction

Behavioural, physiological and cellular processes in the body are timely regulated by an internal time-generating mechanism known as the circadian clock. This endogenous clock produces ~24-hour circadian rhythms (CRs), which ensure optimal organismal adaptation to the time-varying demands of the day/night cycles. CRs are driven at the cellular level by regulatory transcriptional-translation feedback loops consisting of repressor and activating elements [1]. The core-clock and clock-controlled genes (CCGs) are involved in numerous important processes in the body, including regulation of hormonal secretion [2], sleep/wake cycles [3], metabolism [4], memory formation [5] and immunity [6]. The disruption of the CR is associated with various diseases, including Parkinson’s Disease (PD) [7–10].

PD is the second most common neurodegenerative disorder, affecting about 8.5 million people worldwide, according to the latest Global Burden of Disease data released in 2019 [11]. Results from clinical studies and animal models have shown that circadian misalignment is an important feature of PD [7, 12–14], and can be a potential mechanism underlying neurodegenerative progression of the disease. CR alterations have been associated with an increased risk of PD development [15] and are present at early stages of PD [16]. Circadian dysfunction may also be implicated in other PD related symptoms like motor and non-motor symptoms, such as sleep disturbances, where daily fluctuations can occur. As the disease progresses, several PD patients reported more troublesome motor symptoms in the evening hours [17, 18], decreased response to levodopa in the evening doses [19], around 70% of patients suffer from night time akinesia [20]. Conversely some patients described improvement in motor or non-motor symptoms in the morning, the so called “sleep-benefit” [21]. It is therefore essential to characterize the molecular clock, which might be translated into applications to revert the CR changes or optimize the time for certain activities for PD patients, ranging from daily activities to timing of medication intake, in agreement with the individual CR [22, 23].

One putative mechanism for circadian dysfunction in PD is the alteration in the expression levels of core-clock genes. In peripheral blood samples of a large cohort of PD patients (N = 153) and healthy controls (N = 156), a significant reduction of *BMAL1* (also known as *ARNTL*), *CLOCK*, and *PER1* expression was observed [24], and decreased expression of *BMAL1* validated in whole-blood samples (N = 30 patients, and N = 15 controls) collected over a time-course of 24 hours with a 3 hour-sampling frequency [16]. Another recent study monitored the expression of peripheral clock-gene expression from hair samples of PD patients (N = 17) under dopaminergic treatment [25]. Patients with a positive response to evening time bright light therapy showed a phase shift of *PER3* hindering a restoration of CR, which may be correlated with the improvement of sleep symptoms. Another line of evidence of molecular clock dysfunction in PD was reported in a recent study by our group [26] based on an analysis of the circadian clock network [27] in an idiopathic PD cohort. In this analysis, PD patients showed weaker correlations in the expression clock genes, within the network, than in age and sex matched healthy controls. The reduced correlation seen in PD supports a dysfunction in the circadian regulatory mechanisms. Altogether, these data suggest that the transcription of core clock genes may be altered in PD patients. Yet so far, studies included either small sample sizes, heterogenous patient populations or lack time-course data, thus posing an obstacle for the evaluation of a comprehensive molecular characterization based on individual CR. It thus remains to be elucidated if these alterations are associated with different clinical profiles, either in terms of disease stage or of disease progression, or if they contribute to the disabling symptoms patients suffer. In this study, we aim to address such open questions by a detailed characterization of the circadian expression profiles of core-clock genes (e.g., *BMAL1*, *CRY2*, *PER2*, *NR1D2*) and clock-controlled genes (e.g., *IL-6*, *AKT1*) obtained from saliva samples of PD patients and non-PD controls across two consecutive days. This molecular characterization will be further correlated to additional subgroup analysis with respect to clinical traits such as disease progression, and the presence of motor and non-motor symptoms. In addition, we will consider for our analysis different subgroups with respect to sex, treatment dose, presence and fluctuation of motor and non-motor symptoms and other clinically evaluated parameters to compare the changes in CR patterns. By collecting a detailed clinical assessment, we aim to also explore a potential correlation of changes in CR expression with respect to clinical phenotypes that can be used to predict faster or slower disease progression, and for better understanding the role of CR alterations in the molecular mechanisms of PD. In parallel, by collecting sleep data, through questionnaires, actigraphy and polysomnography (PSG), we aim to characterize sleep associated-structural changes that can be associated to malignant PD phenotypes and further correlate those with gene expression data. Additionally, we plan to carry out analysis of neuropathological and clock-pathways using a multiplexed system on a subset of patients based on disease duration, which can reveal novel gene expression alterations. The re-assessment of clinical evaluation as a follow-up may also show if baseline clock gene alterations may be correlated with a faster disease progression after 1 year. In the long term, the knowledge thus gained could be used to predict or monitor disease progression and adjustment of therapies to the CR of each patient.

## Materials and methods

### Aim of the study

The planned study aims at measuring gene expression (via RNA quantification) from saliva time-course sampling, to characterize CR of individuals with PD, and compare it with the CR from healthy (non-PD) controls.

### Study design

**Fig 1:**
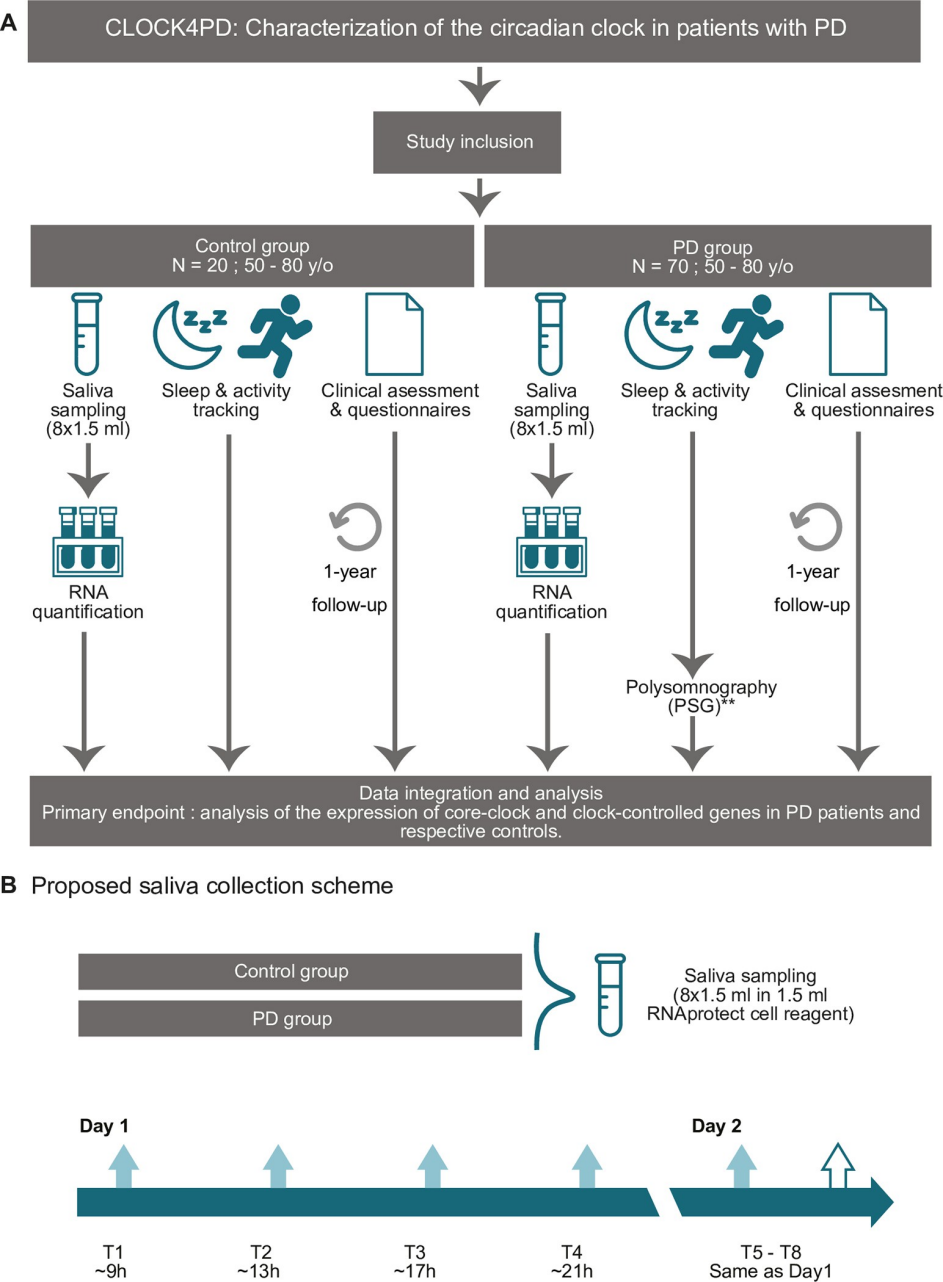
Study design flow chart. **A** Participants are requested to collect saliva, which will be further processed for RNA isolation and quantification of gene expression via RT-PCR or NanoString^Ⓡ^ methodologies depending on the quality and amount of available RNA per sample. Additional data will be collected for activity tracking using a wrist-worn actigraphy device and questionnaires. (**): Actigraphy and questionnaire data will be collected from all patients and controls. From a subset of patients with PD (N = 20 in total) ambulatory polysomnography (PSG level 2) will be carried out. **B** Saliva sampling is carried out according to the proposed scheme. Participants from both the control and the PD group will be advised to collect saliva every 4 hours, during their active/awake hours, across two consecutive days, by their attending physician during the introductory session. The icons used in the figure were sourced from Flaticon (https://www.flaticon.com/), and modified for the better representation.

This is a prospective, monocentric, non-interventional observational study to collect data from controls and individuals with PD, which will be carried out at the CNS-Central Neurológico, in Portugal (**Fig 1**). Saliva samples will be collected by participants using home-kits. Subsequently, clock gene expression profiles for a selected set of genes including *BMAL1*, *PER2*, *CRY2*, *NR1D2*, *AKT1*, *SIRT1*, *IL6*, *PDHB*, will be analysed via Reverse transcription polymerase chain reaction (RT-PCR) and compared between the PD and control group as the primary objective. In addition, sleep & activity will be monitored in both control and patient groups using actigraphy and clinical assessment, as well as questionnaires (see section **Data Collection** for further details). The clinical assessment and questionnaires will be repeated, for patients and controls, in a 1-year follow-up, to assess the disease dynamics. In a sub-group of PD patients (20 in total, with mild/motor dominant and diffuse/malignant phenotype) ambulatory PSG data will be collected. The recruitment of study participants started in July 2022 and is planned until the end of 2024.

### Study objectives

#### Primary objective

To test the hypothesis that the expression and rhythmicity of certain core-clock and clock-controlled genes is altered in patients with PD compared to age- and sex-matched non-PD controls.

#### Secondary objectives

Subgroup analysis to investigate CR variations will be assessed based on clinical traits including biological sex, disease phenotype (mild motor dominance/moderate PD versus diffuse malignant PD with a faster disease progression), presence of motor and non-motor fluctuations, disease duration, questionnaire scores, L-Dopa equivalent dose, cognitive function (based on MOCA assessment). In addition, by performing the detailed clinical assessment for sleep disorder symptoms in PD including overall sleep quality, restless legs syndrome (RLS), rapid eye movement sleep behaviour disorder (RBD), excessive daytime sleepiness, and by collecting actigraphy data we aim at correlating them with changes in gene expression profiles. Using the collected data from diaries (the Scales for Outcomes in Parkinson’s disease -SCOPA- diary) with symptom fluctuations across the day, we aim to explore circadian fluctuations in disease symptoms (such as worsening of the symptoms in the evening), and to correlate such outputs with measured CR changes.

#### Explorative objectives

Investigating the putative correlation between CR and the expression of up to 800 target genes, which will be quantified using a multiplexed gene expression system (NanoString^Ⓡ^ nCounter SPRINT Profiler). The experiment will be carried out on a subset of individuals to compare CR changes in patients with long or short disease duration, versus controls.

### Sample size estimation

A sample size calculation can be carried out based on pilot data or theoretical values originated from similar studies [28, 29]. For this particular study, since there is no pilot data generated exactly using the same protocol (e.g., repeated saliva sampling in PD population) for detection of above-described genes of interest, we used values from literature. Previous studies analysing the expression of clock genes in PD patients have shown alterations in the expression levels of the core-clock gene *BMAL1* (with a 3-hour sampling interval; using a venous cannula to avoid interfering with the regular sleep times of the patients) in blood samples [16, 30]. According to Breen *et al*. [16], expression analysis of peripheral clock genes was carried out with 30 early-stage PD patients versus 15 controls. These patients are described as “newly diagnosed” PD, although the authors do not state the timing since diagnosis required for recruitment. On average, the Hoehn and Yahr (H&Y) scale stage was 1.27±0.45, the motor score in unified Parkinson’s disease rating scale (UPDRS) (part III) was 24±6 and 80% of the patients werebeing treated with an average 312±157 levodopa equivalent daily dose (LEDD). They were taken from a larger cohort that had been diagnosed with PD for 2.2±2.9 years, which represents a population of PD patients with a mild early-stage disease. In this study the absolute values of clock genes expression were not reported. The study by Cai and colleagues [30] was carried out in 17 PD patients and 16 age-matched controls. These patients were on average 62±2.5 years old, had an average motor score in UPDRS of 28.3 ± 4.5. Among those, 4 of the PD patients were drug-naive (no treatment), the remaining 13 were medicated patients receiving a mean dose of levodopa equivalent to 310 ± 58.6 mg (range: 100–700 mg). Although the authors do not state any specific requirements regarding disease severity, these characteristics also suggests a cohort in early PD stages [31]. The results showed [30] differences in the expression levels of the core clock gene *BMAL1* in PD patients versus controls. At baseline (0h) the relative gene expression showed a wider difference (mean, standard deviation): 30.30 ± 5.45 vs 100 ± 24.71 respectively, as compared to 21h, 22.17 ± 4.09 and 51.14 ± 8.31, quantified by RT-PCR. Even assuming this smaller difference, a small sample size of 11 patients is sufficient to detect significant differences in patients and controls [32]. In this project, we further want to explore alterations in gene expression of core-clock and CCGs as our main outcome, and plan to include participants from two extreme phenotypes (mild-motor predominant and diffuse-malignant subtypes) to investigate the predictor role of clock genes in distinct disease phenotypes as an exploratory outcome. A previous study reported ~ 1:3 (35%) prevalence of diffuse malignant PD phenotype in random PD populations [33]. Thus, we planned to recruit 70 PD patients, with a predicted number of 24 malignant phenotypes (~35% of the total PD population) and 20 controls.

### Participant recruitment

In the scope of this study, we plan to recruit 20 healthy volunteers and 70 volunteers with PD.

#### Inclusion criteria (PD group)

1) Idiopathic PD patients with more than 3 years of disease, to reduce diagnostic error and include most patients in moderately severe disorder, with possible motor and non-motor fluctuations

2) Modified Hoehn & Yahr (H&Y) score 2–3. H& Y is a scale which is used to characterize the level of functional disability in PD and assess progression of symptoms [34, 35]. A score of 2 indicates a bilateral involvement without impairment of balance; and 3 indicating mild to moderate bilateral disease; with some postural instability, but the patient is physically independent.

3) Male and female participants with age 50 to 80 years old;

4) Ability to understand and follow study-related instructions;

5) Willingness to participate in the study and provide a signed informed consent form.

#### Exclusion criteria (PD group)

1) No consent to participate in the study. It is not necessary to provide reasons;

2) Presence of Parkinson’s disease related dementia;

3) Previous diagnosis of moderate or severe obstructive sleep apnea (the number of apneas or hypopneas recorded during the study per hour of sleep—AIH>15) if non-compliant to CPAP therapy defined as >70% of the day with ≥ 4h CPAP usage;

4) Modified H&Y ≥ 4. A value of 4 indicates a severe disability though the patient may still be able to walk or stand unassisted, whereas 5 indicates confinement of the patient to bed or wheelchair unless aided.

5) Presence of other neurological disorders besides PD, including stroke, epilepsy, migraine, trigeminal autonomic headaches, inflammatory CNS disorders;

6) Presence of shift work defined as at least one-night shift or early morning shift per week indicating any arrangement of daily working hours other than the standard daylight hours (~7/8 am–~5/6 pm) in last 12 months at the time of recruitment. We also plan to exclude any patient with a previous diagnosis of shift work disorder, according to the International Classification of Sleep Disorders III;

7) Acute infection including oral infections. A chronic infection is characterized by the persistence of the symptoms longer than 6 months, and depicts different progression and symptoms than acute infections [36, 37]. Here we focused our exclusion criteria particularly for acute infections that may impact the saliva collection such as COVID or other viral and bacterial infections, which temporarily may interfere with the circadian profile. Chronic oral infections such as Gingivitis, also known as gum disease, would not be expected to lead to short term alterations, but are rather an integral part of the medical profile of a given patient.

All information regarding any acute or chronic infections (oral or other types) will be recorded during sample collection for all participants. Participants will be advised by their attending physician not to sample in case of any type of infections, during the introductory session.

In this patient population it is common to have people treated with drugs commonly used for PD treatment (Levodopa, dopamine agonists, Catechol-O-methyl transferase (COMT) inhibitors, Monoamine oxidase (MAOI) inhibitors, amantadine), but also benzodiazepines (mostly clonazepam for Rapid Eye Movement (REM) behavior disorder), antidepressants due to the frequent comorbid anxiety or depression, melatonin (for REM behavior disorder or sleep disturbances) and occasionally antipsychotics (for hallucinations and other psychotic symptoms). In this study, we will not interfere with the treatment and management of the patients in the course of sample collection. In addition, due to the regular need for medication intake for PD patients, a medication stop during the full duration of sampling (48 hours) would be very difficult for the patients, and would strongly disrupt their daily routine. In order to have a more representative patient population and also to not seriously limit recruitment, we will include all patients despite medications. All relevant data related to the exact medication, its dosage, and intake timing, during sample collection, will be documented for subsequent analysis, to identify confounding factors that may impact the results. These factors will then be considered through statistical models such as regression, to account for their effects.

#### Inclusion criteria (control group)

A control group will be selected age and sex matched with the patient group. 20 participants will be recruited, with an even distribution of males and females.

#### Exclusion criteria (control group)

1) No consent to participate in the study. It is not necessary to give reasons;

2) Significant sleep disorders as evaluated in baseline visit (V0) and defined as Pittsburgh Sleep Quality Index (PSQI)>5, suspected RLS in RLS questionnaire, suspected sleep apnea in the Snoring, Tiredness, Observed Apnea, Blood Pressure, Body Mass Index, Age, Neck Size, Gender, (STOP BANG) questionnaire, excessive daytime sleepiness defined as Epworth Sleepiness Scale (ESS) ≥10;

3) Presence of shift work defined as at least one-night shift or early morning shift per week indicating any arrangement of daily working hours other than the standard daylight hours (~7/8 am–~5/6 pm) in last three months relative to the time of recruitment; We also plan to exclude any person with a previous diagnosis of shift work disorder, according to the International Classification of Sleep Disorders III;

4) Presence of neurological disorders including movement disorders, stroke, epilepsy, chronic migraine or episodic migraine requiring prophylactic treatment, trigeminal autonomic headaches, inflammatory Central Nervous System (CNS) disorders or neurodegenerative disorders;

5) Presence of serious mental illnesses including major depression, Psychosis, bipolar disorder, generalized anxiety disorder;

6) Presence of a previous diagnosis of sleep disorder, including untreated moderate or severe obstructive sleep apnea (OSA) defined by an AIH (the number of apneas or hypopneas recorded during the study per hour of sleep) >15 in a previous polysomnographic study, chronic insomnia disorder, restless legs syndrome, parasomnias, circadian rhythm sleep disorders. OSA patients treated and compliant with Continuous Positive Airway Pressure Therapy (CPAP), as defined for the patient’s group will be accepted;

7) Acute infection including oral infections. Chronic oral infections such as Gingivitis, will not be considered as an exclusion criterion when the participant is treated and regularly monitored. Information regarding acute or chronic (oral or other types) infections will be recorded during the course of sample collection for all participants. Participants will be advised by their attending physician not to sample in case of any type of infections during the introductory session.

### Data collection

#### Circadian rhythm monitoring from saliva samples

The collection of saliva samples is performed by the participants themselves at home. For this purpose, the participants receive a TimeTeller^®^ kit for the data collection using 3 ml test tubes filled with 1.5 ml of a non-toxic, non-hazardous solution for the preservation of RNA samples (RNA protect Cell Reagent; Qiagen, Cat No./ID: 76526) and 1.5 ml of saliva is required per vial (indicated in the tube with a mark). The participants are advised about sample collection during the introductory session, when they are given the study materials, by their attending physician. On the sampling days, subjects are advised to carry out their normal daily routine and a total of 8 saliva samples should be collected during two consecutive days (e.g., Day1: 4x saliva collection every 4 hours, for example 9; 13; 17; 21 h; Day 2: similar sampling scheme). The samples can be stored at room temperature for a few hours or at 4°C for short-term (~ a week), or at -20°C for long term storage in the fridge of the participants until returned to the study site.

#### Clinical assessment of participants

If PD is suspected in an individual, they are usually referred at an early stage to a specialized center such as the CNS Campus Neurológico and Unidade Local de Saude Santa Maria (ULSSM).

All PD patients and control participants recruited in the course of this study will be subjected to a clinical evaluation that includes:

### Assessment of health status, severity and progression of PD

For this purpose, general demographic and clinical questionnaires will be used to collect information regarding age; biological sex; concomitant medical disorders including hypertension, diabetes, cardiovascular disorders, neoplasm, endocrine disorders, gastrointestinal disorders; concomitant sleep disorders, including sleep apnea, parasomnias, restless legs syndrome, chronic insomnia disorder, medication, equivalent L-dopa dose, symptomatic disease duration;

Supine and 3-min orthostatic blood pressure measurement (Orthostatic hypotension is defined as a drop of 20 mmHg in the systolic blood pressure or 10 mmHg in the diastolic blood pressure from supine to standing positions) will be recorded. For specific symptoms and staging the following assessments will be used:

The Montreal Cognitive Assessment (MoCA)—a brief cognitive screening instrument specifically developed to assess milder forms of cognitive impairment, allowing for evaluation mild cognitive impairment, Alzheimer’s disease, but also cognitive impairment associated with many other clinical conditions including PD [38];

The Movement Disorder Society-Sponsored Revision of the Unified Parkinson’s Disease Rating Scale (MDS- UPDRS)—It was developed to evaluate various aspects of PD including non-motor and motor experiences of daily living and motor complications [39]. The official Portuguese version will be used [40];

Movement Disorder Society Nonmotor Rating Scale (MDS-NMS). The MDS-NMS comprises 52 items, grouped according to clinical content into 13 domains: (1) depression, (2) anxiety,(3) apathy,(4) psychosis, (5) impulse control and related disorders (4 items), (5) cognition, (7) orthostatic hypotension), (8) urinary, (9) sexual, (10) gastrointestinal, (11) Sleep and wakefulness, (12) pain, and (13) other (such as unintentional weight loss, decreased smell, physical fatigue). Items in each category are scored for frequency (from 0 [never] to 4 [majority of time]) and severity (from 0 [not present] to 4 [severe]), which are multiplied to generate the item total score. The MDS-NMS scale also has a new additional Non-Motor Fluctuations (NMF) subscale to assess changes in non-motor symptoms in relation to the timing of anti-parkinsonian medications across 8 domains [41, 42];

Modified Hoehn and Yahr (H&Y)–The Hoehn & Yahr scale is a widely used scale for PD patients that provides an estimate of general clinical function in PD, combining functional deficits and objective signs [34, 35]. It relies mostly in unilateral versus bilateral involvement and postural and gait deficits, to classify disease severity. In its original form it was designed as a 1–5 scale, which was later on refined to include 0,5 steps to provide more discrete information regarding disease severity. This change is referred to as the modified Hoehn & Yahr scale [35]. For our study we will select patients with early and moderately severe PD disorders, including patients with a score of 2 or 3. A patient with a score of 1 have a mild disorder, with unilateral involvement and no axial symptoms or signs. A score of 2 indicates a bilateral involvement without impairment of balance; whereas 3 indicates bilateral disease; with some postural instability but the patient is physically independent. This characterization is based on the neurological evaluation which is performed in a comprehensive way in UPDRS which other of the scales used to characterize the patient population in our study. A scale of 4 indicates a severe disability though the patient may still able to walk or stand unassisted; whereas scale of 5 indicate confinement of the patient to bed or wheelchair unless aided. In accordance with the Movements disorders society recommendations, the H&Y scale is useful for demographic characterization and for exclusion and inclusion criteria definitions. We aim to study patients with more than 3 years of disease duration, to avoid diagnostics errors and, hence, a bilateral involvement is expected. As we are interested in identifying early circadian related changes and to study these as possible markers of progression, we excluded patients with severe advanced PD (H&Y 4 and 5). Furthermore, in this more advanced population, there are many more possible confounders for circadian evaluation, including important lifestyle changes, decreased light exposure, higher prevalence of dementia, which we want to minimize in our population.

### Assessment of sleep and sleep-related disruptions

Epworth sleepiness scale (ESS)– 10 items scale developed to evaluate the propensity to fall asleep/doze off in inappropriate situations [43]. The Portuguese validated version will be used [44];

STOP-BANG questionnaire–a questionnaire that evaluates sleep apnea related symptoms and associated disorders (hypertension, obesity) to access the risk of obstructive sleep apnea. The Portuguese version will be used [45];

PSQI—The Pittsburgh Sleep Quality Index (PSQI) is an effective instrument used to measure the quality and patterns of sleep in the adult. It differentiates “poor” from “good” sleep by measuring seven domains: subjective sleep quality, sleep latency, sleep duration, habitual sleep efficiency, sleep disturbances, use of sleep medication, and daytime dysfunction over the last month [46]. Developers of the original PSQI survey have suggested a cut-off score of 5 for the global scale, which correctly identified 88.5% of the patient group in their validation study [46]. Thus, a PSQI score from 0–4 is commonly categorized as “good” sleep and 5–21 indicating “poor” sleep. Since this filter has been widely used in the field and validated in several studies [47–49], we consider it to be an appropriate threshold for assessment of the results in this study. Different cut-offs can be used in different studies with PD patients to categorize good/bad sleeper categories (e.g., based on a score of <5 or <8 [50, 51]) during downstream analysis. PSQI will be used in the PD study group only to characterize sleep quality. This questionnaire will only be used to exclude subjects in the control group, as we are selecting controls without any sleep disorder. In one of the previous studies [52], in a large-community based study with similar age groups to ours (age [45 – 75]), around half of the population (49.2%) had PSQI score < 5. A sub-grouping of the population according to this cut-off suggested distinct sleep and psychological measures associated with good and bad sleepers validating previously established cut-offs. We are confident that this cut-off will not impair controls selection and it will contribute to have a healthier and stratified control population since PSQI has been shown to correlate well with insomnia and OSA disorders [53]. Thus, we do not expect it to be a limitation for the recruitment. Upon completion of the data, we will also compare the distribution of the PSQI results in our specific cohort and how the results are compared to other population data to potentially consider different cut-offs to categorize the groups further.

SCOPA-SLEEP is a reliable and valid patient-completed instrument for assessing night time sleep (5 items) and daytime sleepiness (6 items) including sleep attacks, in patients with PD [51] that is recommended for the overall assessment of sleep quality [54];

Innsbruck Rapid eye movement sleep behavior disorder (RBD) inventory (RBDI5)–questionnaire with 5 items to evaluate the likelihood of RBD with a sensitivity of 91.4% and a specificity of 85.7% [55];

International Restless Leg Syndrome study group rating scale (IRLS)–scale with 10 items to graduate the severity of RLS symptoms [56];

### Chronotype assessment

Morningness/Eveningness questionnaire (MEQ): MEQ is a self-assessment questionnaire to assess an individual’s chronotype based on their preference for activities during morning or evening. The Portuguese validated version will be used [57];

The ultra-short Munich Chronotype Questionnaire: The (μMCTQ) consists of 6 core-module questions from the original MCTQ [58] that aims to assess a person’s chronotype. The respective questions from the original Portuguese translation of the original questionnaire will be used [59].

The selection of questionnaires aims at evaluating PD characterization and dynamics over time (MDS-UPDRS, H&Y, MDS-NMS, MOCA); sleep quality and sleep related symptoms (PSQI, ESS); sleep disorders and its severity (STOP-BANG, IRLS, RBD I5, ISI), PD specific sleep complaints (SCOPA-Sleep) and chronotype characterization (MEQ, μMCTQ). On average, the interview to perform the above-mentioned assessments takes ~1.5 h and it is also planned to given the subjects enough time to go through self-report questionnaires that will be later reviewed together with the clinical investigator. The total time allocated for interviews and questionnaires includes as well the routine anamnesis during the clinical visits. Additional questions related to health and PD-status, carried out by the attending physician will be performed as a normal “conversation” with the patient, and will be useful to also monitor other important aspects of PD progression like for example the speech ability. For these reasons, we don’t anticipate recruitment or compliance problems based on this part of the protocol.

#### Revised SCOPA diary card

The diary aims to evaluate the fluctuation of motor and non-motor symptoms through-out the day over a two week period [60]. The aim of this analysis is to capture the clinical phenomenon of morning akinesia, sleep benefit or evening akinesia in an objective way, and to be able to correlate this with clock genes dysfunction. The diary evaluates and grades severity of symptoms in 7 intervals over the day (every 3 hours during the day, and during 6 hours at night) and it evaluates 3 dimensions: Mobility, Physical Functioning and Psychological Functioning.

#### Polysomnography (PSG)

A sub-group of 20 PD patients and 20 controls will undergo ambulatory PSG (Somnoscreen plus PSG system) with the following variables: 6 EEG electrodes (F3, F4, C3, C4, O1, O2, referenced to ipsilateral mastoid), right and left electrooculogram, EMG (mentalis), nasal cannula; oro-nasal thermistor, respiratory and abdominal muscle bands; oximetry; flexor digitorum brevis EMG bilaterally.

#### Medical history

Data relating to PD subjective symptoms; comorbid conditions and medications will also be documented by the medical staff using Case report form (CRF). All information concerning name, dose and timing of intake of medication, during the time of the clock genes measurements will be recorded.

#### Activity measurements

Upon recruitment patients and control subjects will be provided a wrist-worn actigraphy device (AX3, Axivity) which will be used to track resting and activity cycles. The participants will use the device over 15 days and saliva samples will be collected during these days.

#### Progression evaluation

All measurements will be repeated at 1 year follow-up in PD patients, including clinical assessment, questionnaires and scales, and movement tracking with actigraphy. Global composite outcome (GCO): During follow-up measurements, which will be collected a year after GCO will be assessed as suggested in a previous study [33].The severity of the disease will be evaluated considering 4 mains domains: 1. Motor symptoms: sum of UPDRS–Part II and UPDRS–Part IV scores. 2. Motor signs: UPDRS–Part III score. 3. Cognition: graded as normal (score = 0), single-domain Mild Cognitive Impairment (MCI) (score = 1), multiple-domain MCI (score = 2), mild-to moderate dementia (score = 3), and severe dementia (score = 4). 4. Other nonmotor manifestations: equal weighting of standardized (0–4) scores for depression, anxiety, hallucinations, apathy, somnolence, insomnia, orthostatic dysfunction, urinary dysfunction, and constipation. Increased dopaminergic medication will also be considered. A GCO will be then created by merging the standardized scores for these 5 categories. The total score will be calculated by summing up the quintile values of different domains (range, 0–16).

#### RNA quantification with NanoString^Ⓡ^ nCounter SPRINT profiler

Additional clock and clock-related genes will be measured on a subset of participants (N = 3 male patients with longer (>15 years) duration of PD, N = 3 male patients with shorter (<5 years) disease duration and N = 6 controls with 3 females and 3 males to assess potential sex-bias). Homo Sapiens Neuropathology Panel will be used for this experimental setup which includes 770 genes linked to six core mechanisms altered in neurodegeneration: neurotransmission, neuron-glia interactions, neuroplasticity, preservation of cellular structure, neuroinflammatory responses, and metabolic processes respectively. The curated list of genes are involved in activated microglia, angiogenesis, apoptosis, autophagy, axon and dendritic structure, carbohydrate metabolism, chromatin modification, cytokines, growth factor signalling, lipid metabolism, matrix remodelling, myelination, neuronal connectivity, neuronal cytoskeleton, oxidative stress, tissue integrity, transcription &splicing, transmitter release, transmitter response and reuptake, transmitter synthesis and storage, traffic factors, unfolded protein response and vesicle trafficking pathways. In addition, NanoString^Ⓡ^ Human Neuropathology Panel also includes a set of house-keeping genes (e.g., *GUSB [61], TBP [61], PGK1 [62]*) and our custom design panel for core-clock and clock-controlled-genes (in total 55 genes) include the housekeeping genes *GAPDH* and *ALAS1 [63]*. Using this data, we can estimate a variation value to apply as an additional normalization factor if required also to complement our analysis with respect to RT-PCR data. To further investigate the changes in the CR, a custom designed panel with 55 unique genes including a published set of extended-core clock network [27], and other clock-regulated genes from a recent clock-model [64] will be used.

### Outcome measures

#### Primary outcome measure

Assessment of core-clock and clock-controlled gene expression profiles in saliva samples obtained from PD patients and healthy controls.

#### Secondary outcome measures

Based on the obtained clinical data, subgroup analyses will be carried out. These will include biological sex, presence of motor and non-motor fluctuations, disease duration, questionnaire scores, L-Dopa equivalent dose, cognitive function (based on MOCA assessment).

To determine the potential changes with respect to disease phenotype, a sub-group analysis will be carried out between mild-motor dominance/moderate PD versus diffuse malignant PD with a faster disease progression. For the subgroup of patients submitted to PSG we will further analyse the potential correlation of observed CR changes with sleep macro and micro-structural changes. In addition, the actigraphy data will be collected from all patients and controls to assess overall sleep quality and latency (such as time spent in bed prior to falling asleep) will be correlated to the gene expression profile of clock-controlled genes. It is also planned to investigate possible correlations of overall disturbances in activity cycles with relative ratios of clock genes using a correlation analysis.

#### Explorative outcome measures

Additional clock and clock-related genes will be measured with a NanoString^Ⓡ^ nCounter SPRINT Profiler, for a subset of participants,using Homo Sapiens Neuropathology and a custom designed panel of clock-controlled genes.

#### Data management

The study team will archive the source documents for each participant, which consists of the informed consent forms, saliva collection sampling form, questionnaires, clinical assessment reports, self-report diaries, data from actigraphy device and gene expression results (CT values). The study will be carried out in accordance with the applicable data protection regulations. The results will be published in anonymized way so that it will not be possible to draw any conclusions regarding the identity of the subject. The re-identification list consists of pseudonymous code assigned for each subject, original copies of the participant records, informed consent forms and associated clinical data will be archived for a 10-year period.

### Safety considerations

Our study is a non-interventional, observational study therefore a safety consideration plan is not applicable.

### Statistical analysis plan

#### Descriptive analyses

Descriptive statistics will be evaluated for demographic information and clinical variables such as age, sex, cognitive impairment (MoCA), PD-related motor fluctuations (MDS-UPDRS-part IV), the MDS-UPDRS items and the H&Y stage. All continuous variables will be summarized using the following descriptive statistics: n (non-missing sample size), mean, standard deviation, median, maximum and minimum.

#### Analysis for primary endpoint

The expression values of the individual clock genes of all subjects, measured by RT-qPCR and/or NanoString^Ⓡ^, will be evaluated using the ddCT method and log counts respectively, each gene normalized to the mean value of all time points for each individual and pre-normalization to a housekeeping gene (GAPDH) will be carried out. Since the *GAPDH* expression is also measured over the time-course in our experimental design (across 2 consecutive days, every 4 hours except during the night time), the RT-PCR data will first be normalized according to the overall expression of GAPDH thus, we do not expect such variation to influence the interpretation of the results. A heterogeneous mix of epithelial and leukocyte cells has been previously reported to be present in saliva samples [65]. Notably, though saliva exhibits lower epithelial cell content in both children and adults compared to buccal swabs [65]. The between-subject variation in epithelial cell content was higher in saliva samples for both age groups, possibly due to variations in oral inflammation. The exact variation of specific groups of cells across individuals is not defined, however we do use reference genes (e.g., *GAPDH*) in our analysis as indicated. In addition to a pre-normalization of the data to a reference gene, we normalize each gene expression value (e.g., Time point 1) to the average expression across the time-course of each individual, these different steps should contribute to reduce biological background and allow for interindividual comparisons. We have tested this method previously and obtained reproducible results, which also lead to comparable conclusions regarding circadian rhythms, when using different types of biological samples [66]. The temporal expression profile of each clock gene for each subject is tested individually for circadian rhythmicity using harmonic regression analysis. For this, the model y(t) = m + acos(ωt) + bsin(ωt) is fitted to the data. CR parameters (phase, amplitude, mesor) for the sub-groups according to clinical traits will be estimated using harmonic regression with 24 hour fixed period. Lack of significant CR (the null hypothesis, H_0_ = 0) will be tested against the existence of a circadian gene expression profile (the alternative hypothesis, H_A_≠0). Rhythmicity will be tested by commonly used approaches in the field such as F-test as described by Cornelissen and colleagues [67]. According to our own experience from previous studies [66, 68, 69] including healthy participants or leukemia patients sampling of saliva is not a problem. In PD, sialorrhea is a frequent complaint, affecting more than 80% of the patients [70] and thus this sampling method is unproblematic. On the contrary, a few patients, complain of xerostomia [71], which might constitute a problem for our sampling method. Despite this occurrence, previous studies have used saliva samples in PD patients and report very infrequent failures to collect saliva. Figura *et al*. [72], report 2 PD patients out of 26 that were not able to collect saliva. The patients included in this study are similar to the ones we will evaluate, but the saliva sample required on the study of Figura *et al*. 10 ml of saliva, which is much more difficult to obtain than the 1.5 ml required per vial in our study. Importantly, in this study, 9 out of 26 controls could not collect saliva which further informs about it constituting a more difficult saliva collection method [72]. Other study, where PD patients were required to collect 2-ml of saliva, reported no patients excluded due to inadequate saliva samplings [73]. We thus anticipate that saliva collection will be feasible in our study population. Nevertheless, to account for possible missing samples, we provide 8 sampling tubes with each sampling round. A minimum of 4 samples per day within two consecutive days, is required for successful assessment of the circadian oscillation of gene expression, and thus will be considered for the rhythmicity analysis. The differences in CR and mean gene expression will then be evaluated by CircaCompare [74] and Limorhyde [75] or equivalent algorithms. The study is considered successful if circadian expression profiles can be detected in clock genes from saliva samples of PD patients and controls.

#### Analysis for secondary endpoints

Sleep disturbances: 1) Sleep quality: Patients will be divided into those with significant sleep complaints based on the SCOPA-SLEEP questionnaire. Poor sleepers will be considered if SCOPA-Sleep NS subscale > 3 OR SCOPA-Sleep DS > 4 [51]. Statistically significant differences between control and PD patients with and without sleep complaints at all time points will be evaluated; 2) RBD: Patients will be categorized as having or not having RBD, based on the Innsbruck RBD inventory>0.25 [55]. Statistically significant differences between control and each PD patients’ group at all time points will be evaluated; 3) RLS: Patients will be divided into two groups, based on the presence or absence of RLS as determined by a clinical interview; the International Restless-legs syndrome Study Group Scale (IRLSGRS) will be used to characterize the severity of symptoms in patients and controls. Statistically significant differences between control and PD patients with and without RLS symptoms at all time points will be evaluated; 4) Excessive daytime sleepiness: Patients will be divided into two groups, based on the presence (ESS ≥ 10) or absence of excessive sleepiness (ESS < 10). Statistically significant differences between control and each PD patients’ group at all time points will be evaluated. We further plan to correlate changes in circadian gene expression

Sleep microstructure: In the scope of this study, we plan to recruit a total of 20 PD patients and 20 controls. PSG data including microstructural sleep variables such as sleep EEG delta power, spindles density, beta power, REM without atonia index, rapid eye movement density and small sleep spikes presence will be collected from these patients. These PSG variables will be correlated to clock gene expression and, in addition, using the follow-up clinical assessment, it is planned to evaluate these changes as a predictor of the progression outcomes.

Motor fluctuations: Patients will be categorized as having or not having motor fluctuations based on the responses to MDS-UPDRS part IV. Patients with no motor fluctuations must answer 0 to all questions. Statistically significant differences between control and PD patients with and without fluctuations at all time points will be evaluated.

Non-motor fluctuations: Patients will be categorized as having or not having non-motor fluctuations based on the MDS-NMS Non-Motor Fluctuations (NMF) Subscale. Patients with no non-motor fluctuations must answer 0 to all questions. Statistically significant differences between control and each PD patients’ group at all time points will be evaluated.

End of day motor worsening: Data from the Revised SCOPA Diary Card for PD will be used to identify patients with evening worsening of motor symptoms. Statistically significant differences between control and each PD patients’ group at all time points will be evaluated. In parallel, activity data and cortisol data will be collected, it is planned to correlate these measurements to gene expression data with a potential influence on end of day worsening of motor symptoms.

Morning akinesia: Data from the Revised SCOPA Diary Card for PD will be used to identify patients with morning worsening of motor symptoms. Statistically significant differences between the control and each group of PD patients’ at all time points will be evaluated.

Rest-activity cycle: The rest-activity cycle measured through actigraphy, is one of the most common clinical correlates of the circadian rhythm. PD patients show decreased activity during the day and higher movement levels during the night, correlating with an overall flattening of the circadian curve [76–79]. Whether these changes are due to intrinsic circadian rhythm dysfunctions or the result of extrinsic factors, like bradykinesia, sleep disturbances and drug effects is currently unknown. Several parameters such as total sleep time (TST), sleep efficiency (SE), sleep latency, wake after sleep onset (WASO), and number of awakenings during sleep will be assessed through actigraphy. Other circadian parameters that will be evaluated through actigraphy data include: 1) Inter daily stability (IS) which aims at quantification of the stability of rest-activity rhythms and/or the invariability across different days. Thereby, IS will provide information regarding the coupling of the rhythmic activity to external timing cues (such as daily light cycles), which entrain an organism’s internal circadian clock to the earth’s 24 h periodic cycle. The obtained IS value will vary between 0 (indicating Gaussian noise) and 1, with values closer to 1 indicating stronger coupling; 2) Intra daily Variability (IV), which is used to assess the fragmentation between rest-activity patterns. IV approaches approximately zero in a perfect sine wave and reaches to two for Gaussian noise; 3) Relative amplitude (RA) can be measured based on the M10 (the ten hours with maximal activity) and L5 (the five hours with minimal activity) values. Generally, in humans, M10 consists of 10 h during the day but can be influenced due to, for example, daytime napping. L5 reflects the movement during the night, as well as indicator of other sleep disturbance such as arousals and awakenings.

In parallel, actigraphy data will be collected, which will be correlated to the peak time of sleep/activity cycles and to the peak time of expression for the assessed clock-controlled genes. Among these, for clock genes which show a significant correlation, the difference in terms of circadian expression profiles between patients and controls will be investigated. In addition, we plan to construct a rest–activity rhythm index (RI) using a Harmonic Hidden Markov Model (HHMM) developed by Huang and colleagues [80]. HHMM incorporates a 24-hour harmonic oscillator into the dynamic Markov process in order to model various activity states (such as high, or low activity) and was shown to detect the alterations in rest-activity patterns over the course of chemotherapy in cancer patients [80], and more recently for obesity patients [81] using actigraphy data. By using this approach, we expect to capture the alterations in the rest-activity patterns among PD patients versus controls. Subsequently, we plan to correlate the results with the observed changes in gene expression levels.

Medications: Levodopa equivalent dose (which combines in one variable the different dopaminergic medications) will be correlated to the expression of clock genes and included in multivariate models as a possible confounder. We also plan to evaluate the association between treatment with benzodiazepines, antidepressants, melatonin and antipsychotics and the expression of clock genes.

#### Missing values and outliers

The imputation of missing values will be avoided and an outlier detection will be carried out. The outliers will be then retained or marked as missing according to the decision of the data management team.

### Ethics statement

This study was reviewed and approved by the ethics committee of Medical School Hamburg (Ethics approval: MSH-2021/178) and CNS-Campus Neurológico (Ethics approval: CNS-3-2022). The participants provided their written informed consent to participate in this study.

### Status of the study and timeline of the study

Recruitment started upon ethics approval in March 2022. The recruitment and data collection are planned to be carried out until December 2024.

## Discussion

PD has a profound impact on the life of affected individuals, and places a substantial burden on healthcare and economy, a challenge that is poised to grow given the increase in life expectancy and is estimated to reach over 12 million cases globally by 2040 [82]. The lack of a definitive cure or reliable diagnostic criteria underscores the need for the development of novel biomarkers to enhance early detection, accurate diagnosis, and effective monitoring of disease progression [83]. Studying the interplay between CR alterations in PD patients holds a significant relevance not only due to the need for development of novel biomarkers for PD, but to also provide valuable insights for management of disease symptoms, heterogeneity or progression. Sleep/wake disturbance for example, is an early non-motor symptom observed in PD [84], which about 80% of patients suffer according to a systematic review [85]. CR alteration of blood pressure [86, 87], core body temperature [88], hormonal regulation (e.g., melatonin and cortisol) have been observed in PD patients pointing to an overall disruption in the circadian system. At the molecular level, a loss of circadian expression of core-clock genes such as *BMAL1* in peripheral blood mononuclear cells [16], l leukocytes [30] and fibroblasts of PD patients [89] were observed. Although these studies shed light on CR disruption on PD, at the molecular level a comprehensive characterization is still missing. To bridge this gap, we aim to evaluate core-clock and clock-controlled gene expression profiles from saliva samples obtained from PD patients and respective controls. CR are known to vary with sex [90], and across the life-span [91]. In this study, by including controls in the same age range as the PD patients, we aim to overcome the limitation of an age difference. To further characterize potential sex-dependent differences, we will carry out a sub-group analysis between females and males. Using the comprehensive clinical data collected, we plan to carry out additional subgroup analysis to determine clinical features that might influence the CR. Data from this study will contribute to a comprehensive characterization of CR differences in individuals with PD with respect to healthy controls, and will be correlated to additional data collected from questionnaires, diaries and activity tracking. Our study further aims to explore alterations in PD sub-group of patients with diffuse malignant and intermediate/mild-motor predominant phenotype. For this sub-group analysis ambulatory PSG data is additionally planned to be collected, which will allow for further characterization of sleep metrics. and can be correlated to gene expression changes. We further plan to carry out a sub-group analysis with respect to follow-up clinical assessment, which will take place 1-year after recruitment to correlate gene expression changes with disease progression, which can be used as a predictor of disease phenotype.

PD progression is reported to be dependent on the disruption in molecular pathways associated to mitochondrial dysfunction, impaired protein degradation and aggregation, oxidative stress, inflammation, apoptosis and cell death [92, 93] in which the circadian system is involved. In our recent study [26] we observed similar gene expression changes (up-or-down-regulation) due to the knockout of core-clock genes (*BMAL1*, *PER2*, *NR1D1*) and PD patients versus healthy controls. Both conditions showed striking changes in genes involved in immunity and inflammation, extracellular matrix organization and focal adhesion, oxidase activity and endocytosis, axonal guidance and structure, cell cycle and growth, autophagy, cell death and mitochondrial function, providing further evidence of the possible role for the dysregulation of the circadian clock in PD related mechanisms of neurodegeneration. In the course of this study, as an exploratory analysis we will use NanoString^Ⓡ^ multiplex system to characterize alterations in CR expression of genes involved in several of these neuropathology-related pathways (770 genes) to identify new targets. For this purpose, we plan to short-list a group of patients with a longer and shorter disease duration and compare to age-matched non-PD controls. While the sample size employed in this study provide valuable insights, applying these results to broader and more heterogenous populations will require studies with larger populations. Additional patient stratification factors may further contribute to understand the CR changes in diverse groups, and be used for optimization of treatment methods. The findings of our study can be in this way translated to other PD populations and be used for the identification of optimal intervals for activities such as sleep or physical exercise to improve management of PD.
